# Menstrual and Reproductive Factors and Risk of Lung Cancer among Chinese women, Eastern Gansu Province, 1994-1998

**DOI:** 10.2188/jea.13.22

**Published:** 2007-11-30

**Authors:** Alina V. Brenner, Zuoyuan Wang, Ruth A. Kleinerman, Shujie Lei, Catherine Metayer, Wenlan Wang, Jay H. Lubin

**Affiliations:** 1 Division of Cancer Epidemiology and Genetics, National Cancer Institute, 6120 Executive Boulevard, Rockville, EPS, Bethesda, MD 20892, USA.; 2 Laboratory of Industrial Hygiene, Ministry of Public Health, 2 Xinkang Street, Deshengmenwai, Beijing 100088, People’s Republic of China.

**Keywords:** female, lung neoplasms, risk factors, reproductive history, menstruation

## Abstract

There are suggestions that women have higher relative risks of lung cancer than men given similar levels of tobacco exposure, implying that sex-related hormones may influence lung cancer risk. We evaluated the association between lung cancer risk and menstrual and reproductive factors on a subset of self-responding females in a population based case-control study in Eastern Gansu Province, China, 1994-1998. The analysis included 109 incident lung cancer cases and 435 controls selected from the census list and frequency matched to cases on age and prefecture. Odds ratios were lower for later ages at menarche (trend, P = 0.015) and later ages at menopause (trend, P = 0.074). Despite limitations, these findings suggest a possible role of hormones in the etiology of lung cancer in females.

There are suggestions that women may have a higher relative risk of lung cancer than men given similar levels of tobacco exposure.^1^^-^^4^ Also, it appears that worldwide lung cancer is more common among lifetime non-smoking women than non-smoking men.^5^^,^^6^ If true, this implies that women may be more susceptible to lung cancer and sex-related hormones may influence its risk.

The lung is usually not considered a target organ for sex hormones; however, specific estrogen and progesterone receptors are distributed in normal lung tissue and some lung cancers.^7^^-^^10^ Animal experiments have demonstrated that sex hormones directly alter fetal lung differentiation^11^ and have affected the incidence of pulmonary neoplasms in adulthood.^12^ In addition, estrogens have been shown to influence the development of carcinoma in other organs such as pancreas,^13^ stomach^14^^,^^15^ and colon.^15^ Epidemiologic data on the role of female hormones in lung cancer etiology have been mixed. Several studies have demonstrated that the use of hormone replacement therapy was associated with an increased risk of lung cancer.^16^^-^^18^ Further, some menstrual and reproductive factors including age at menarche and natural menopause, length and total number of menstrual cycles have been associated with lung cancer, primarily adenocarcinoma, but the patterns are inconsistent.^17^^-^^22^ Finally, one prospective study has found clusters of cancers of the reproductive system and the lung in families, implying possible common risk factors.^23^

The present investigation analyzed the association between the risk of lung cancer and a variety of menstrual and reproductive factors, using interview data from a population-based case-control study conducted in Eastern Gansu Province, China. The study area is predominantly rural, and subjects have little exposure to industrial sources of air pollution and a low prevalence of smoking, thus minimizing the influence of major confounders for lung cancer.

## MATERIALS AND METHODS

### Case ascertainment

We conducted a population-based case-control study of incident lung cancer in males and females in Pingliang and Qingyang, two rural prefectures in Eastern Gansu Province, China, described in detail elsewhere.^24^ Briefly, there were 230 incident lung cancer cases of females, aged 30-75 years, diagnosed between January, 1994, and April, 1998. Cases were identified retrospectively (1994-1995) and prospectively (1996-1998) from two prefecture hospitals, a company hospital located at a nearby oilfield, 15 county hospitals, and local clinics. We also reviewed records from special anti-tuberculosis reporting stations in the prefectures and large near by cities, such as Lanzhou, Xi’an, and Yinchuan for lung cancer diagnosed in residents of the two prefectures. All lung cancer diagnoses were reviewed by an expert panel that consisted of two oncologists, two radiologists, and one pathologist. The panel considered all the available diagnostic evidence for the particular case, including medical records, radiological images, pathology reports and/or slides.

We excluded 118 deceased cases whose information derived from surrogate interviews, in order to ensure accuracy of the reported information. Additionally, three female cases could not be interviewed in person and were excluded, resulting in 109 cases. Histological confirmation was available for 38 out of 109 living cases, a confirmation level consistent with other Chinese studies.^25^ Of these, 16 were small cell carcinomas, 14 were squamous cell carcinomas, 1 was adenocarcinoma, and 7 were characterized as pulmonary neoplasms without any additional specification.

### Control selection

Controls were randomly sampled from the population census list of the two prefectures and frequency matched to cases by gender, within 5-year age group and prefecture in a ratio of 2:1.^24^ A total of 455 female controls were eligible. Fourteen controls were deceased at the time of the interview and six could not be interviewed. Since controls were frequency matched on age, all 435 interviewed controls were included in the analysis.

### Interview

Personal interviews were conducted by trained interviewers using a structured questionnaire. The section on menstrual and reproductive factors included information on age at menarche, length of menstrual cycle, number of days of menstrual flow, number of pregnancies, age at first live birth, parity, age at menopause, type of menopause, use of oral contraceptives, history of irregular menstrual cycles, and type of gynecological surgeries. Data were also collected on sociodemographic characteristics, amount of time spent at home, housing characteristics, lifetime smoking habits, including smoking by other members of the household,^24^ type of fuel used in homes, diet and cooking practices, occupation, pesticide use, and medical histories.

### Statistical analysis

Odds ratios (OR) were used as the measure of association between lung cancer and risk factors of interest. Unconditional, multivariate logistic regression models were carried out using the SAS program^26^ to adjust ORs for confounding factors (reference age, prefecture, socioeconomic status, active smoking, environmental tobacco smoke among never-smokers, the amount of coal used in homes for cooking and heating, cooking oil use, and previous pulmonary diseases). Reference age was defined as the age at diagnosis for a case and the age at interview for a control. A woman was considered post-menopausal if she did not have regular periods for the last 12 months. Assuming an equal period of postpartum amenorrhea, the total number of menstrual cycles up to the reference date was calculated as:

T = [(A1 - A2) × 365 - B × 280] / C,

where A1 = age at last/most recent menstrual period;

A2 = age at menarche;

B = number of live births;

C = average cycle length.

## RESULTS

Table 1 summarizes the sociodemographic characteristics and exposure to tobacco smoke of the participants. Subjects were comparable on reference age (although there was a slightly higher proportion of younger cases), prefecture, and marital status. Cases had higher socioeconomic status as measured by a higher proportion of education above the primary level, a higher income, and greater ownership of goods such as a color television set. There was no excess risk of lung cancer among smokers (ever smoker vs. never smoker), but the proportion of smokers (10%), as well as the amount (mean = 4 cigarettes per day), duration of smoking (mean = 17 years) was low by Western standards. Finally, cases tended to have higher exposure to environmental tobacco smoke during childhood than controls, although the difference was not significant.

**Table 1.** Comparison of female lung cancer cases and controls by sociodemographic characteristics and exposure to tobacco smoke, Gansu province, China, 1994-1998

Characteristic	Cases^1^	Controls^1^	OR	95% CI	p value^2^
	(n=109)	(n=435)			
Age, years					
<45	21	51	1.0	Reference	0.245
45-54	38	162	0.57	0.31-1.07	
55-64	34	144	0.58	0.30-1.09	
65+	16	78	0.50	0.24-1.07	

Prefecture					
Qingyang	48	180	1.0	Reference	0.903
Pingliang	61	255	0.97	0.63-1.50	

Education					
Primary or less	95	415	1.0	Reference	0.019
Tech/vocation	14	18	3.0	1.40-6.49	
College and above	0	2	NA		

Income, yuan/year					
< 2,000	21	106	1.0	Reference	0.107
2,000-2,999	15	86	0.96	0.46-2.01	
3,000-4,399	32	110	1.7	0.88-3.15	
4,400+	41	125	1.8	0.98-3.24	

Number of color TVs					
None	71	352	1.0	Reference	<0.001
At least one	38	81	2.5	1.53-3.94	

Marital status					
Married	94	371	1.0	Reference	0.814
Widowed	15	64	0.92	0.47-1.80	

Smoking status					
Never smoker	98	389	1.0	Reference	0.981
Ever smoker	11	45	1.0	0.50-2.05	

Environmental tobacco smoke in childhood, pack-years					
<1	30	153	1.0	Reference	0.149
1-9	41	159	1.3	0.76-2.17	
10+	16	39	2.0	1.00-4.19	

^1^ Numbers may not add up to column totals because of missing data.

^2^ Test of independence, adjusted for reference age and prefecture.

OR: odds ratio, CI: confidence interval.

Table 2 summarizes the relationships between a variety of menstrual and reproductive factors and lung cancer risk. ORs for lung cancer were lower for later ages at menarche (trend, p = 0.015) and later ages at natural menopause (trend, p = 0.074). ORs were elevated for later ages at first live birth, although the trend was not significant (trend, p = 0.980). There was little suggestion of an association between lung cancer and length of the menstrual cycle, days of menstrual flow, number of live births, and total number of menstrual cycles up to the reference date. Adjustment for a variety of variables (socioeconomic status, active smoking, environmental tobacco smoke among non-smokers, amount of coal, cooking oil use, and previous pulmonary diseases) did not alter these results (not shown). The use of oral contraceptives, as well as history of gynecological surgeries, was rare (less than three percent) in the study population and could not be analyzed.

**Table 2.** Odds ratios and 95% confidence intervals for developing lung cancer accoding to the menstrual and reproductive history among females, Gansu province, China 1994-1998

Menstrual/ reproductive characteristics	Cases (N=109)^1^	Controls (N=435)^1^	OR^2^	95% CI	p for trend
Age at menarche, years					
<15	40	112	1.00	Reference	0.015
15-16	39	188	0.52	0.31-0.88	
17+	28	130	0.54	0.31-0.96	

Age at natural menopause, years^3^					
<45	16	60	1.00	Reference	0.074
45-49	37	136	0.95	0.49-1.86	
50+	11	94	0.39	0.17-0.92	

Average length of the menstrual cycle, days					
<28	21	102	1.00	Reference	0.560
28-29	55	174	1.55	0.88-2.72	
30+	27	145	0.91	0.48-1.71	

Average length of the menstrual flow, days					
≤3	20	75	1.00	Reference	0.690
4-5	62	254	0.94	0.53-1.66	
6+	20	95	0.84	0.41-1.70	

Number of pregnancies^4^					
1-2	14	49	1.00	Reference	0.450
3-4	41	151	1.09	0.54-2.20	
5-6	37	129	1.23	0.58-2.61	
7+	16	102	0.69	0.28-1.68	

Age at first live birth, years^4^					
≤18	22	124	1.00	Reference	0.980
19-22	63	225	1.53	0.89-2.64	
23+	23	82	1.46	0.75-2.84	

Number of live births^4^					
1-2	23	74	1.00	Reference	0.833
3-4	45	196	0.83	0.46-1.50	
5-6	29	103	1.14	0.58-2.24	
7+	11	58	0.81	0.34-1.95	

Number of menstrual cycles up to the reference date					
<300	21	70	1.00	Reference	0.809
300-349	21	94	0.86	0.42-1.74	
350-399	33	103	1.32	0.66-2.63	
400+	25	134	0.79	0.38-1.61	

^1^ Numbers may not add up to column totals because of missing data.

^2^ ORs adjusted for reference age and prefecture.

^3^ 2 cases and 4 controls in the surgical menopause and 43 cases and 141 pre-menopausal controls were excluded from the analysis.

^4^ 4 nulliparious controls and 1 case with missing data were excluded from the analysis.

## DISCUSSION

In this population-based case-control study of lung cancer in a rural area of China, we found a statistically significant decrease in lung cancer risk with later ages at menarche and a marginally significant decrease in lung cancer risk with later ages at natural menopause. ORs for later ages at first live birth were elevated, but the trend was not significant.

Several methodological issues arise in interpreting our results. The present analysis was limited to self-responders which raises the possibility of selection bias (while minimizing the possibility of reporting bias by excluding next-of-kin responders for deceased cases). We evaluated the possibility of selection bias by conducting a survival analysis for all 230 cases based on time from disease incidence to death. None of the variables (socioeconomic status, active smoking, environmental tobacco smoke, amount of coal, cooking oil use, history of previous pulmonary diseases) was a significant predictor of the time from diagnosis to death, after adjustment for calendar-year of diagnosis, age at diagnosis of lung cancer, and prefecture. This suggests minimal selection bias. There was also concern about accuracy of lung cancer diagnosis, since histological verification was available for only 35 percent of the cases. The survival time did not depend on the availability of pathological evidence (about 1 year for confirmed and suspected cases) and results remained unchanged when the data were limited to the pathologically confirmed cases. Residual confounding is always possible. Adjustment for active smoking and restricting data to never smokers, and adjustment for a variety of known confounding variables had no effect on the risk estimates. However, because of limited statistical power, our results should be interpreted with caution.

The strengths of the present study include a population-based design, low prevalence of smoking among women, rural population with limited exposure to industrial pollution, and detailed information on a variety of risk factors for lung cancer.

Overall, there have been few epidemiologic studies conducted on reproductive and menstrual history and risk of lung cancer (Table 3). The results have been inconsistent among the studies and relative to a pattern one might expect based on breast cancer data. One U.S. study showed an increased risk with late menarche^18^ in contrast to our findings. Three studies^17^^-^^19^ suggested an increase in the risk of lung cancer with later age at natural menopause, whereas two others,^18^ including the present study, found an inverse association with age at natural menopause. Ours was the only study to suggest an increased risk with later ages at first live birth, while another study demonstrated an increased risk with number of live births.^22^ Finally, one study demonstrated an association of adenocarcinoma and shorter menstrual cycles^19^ and another reported that cases with squamous cell lung cancer had a higher lifetime number of menstrual cycles than controls, but cases with adenocarcinoma had a shorter period of menstrual flow.^21^

**Table 3.** Summary table of case-control studies on selected menstrual and reproductive factors and lung cancer risk

Referent study		Study area	Cases/Controls	Findings	Adjustment factors
(17)	Taioli E, Wynder EL, 1994^1^	8 cities, USA	180/303	Age at menopause, years (≤40, 41-49, 50+): 0.3 (0.1, 0.8)^2^, 0.8 (0.5, 1.4), 1.0	Smoking, age at diagnosis, years of education, body mass index, type of menopause (natural or surgical, radiation)
(18)	Wu AH, Yu MC, Thomas DC, et al., 1988^1^	Los Angeles County, USA	336/336	Age at menarche, years (≤11, 12, 13, 14, 15+): 1.0, 1.1 (0.6, 1.9), 1.4 (0.8, 2.4), 1.7 (0.9, 3.1), 1.3 (0.7, 2.5) p for trend = 0.13	Pack-years of smoking, years since smoking stopped, and depth of inhalation
				Age at natural menopause, years (≤48, 49-51, 52+): 1.0, 1.0 (0.4, 2.3), 0.6 (0.3, 1.4) p for trend = 0.26	
(19)	Gao YT, Blot WJ, Zheng W, et al., 1987	Shanghai, China	672/735	Age at natural menopause for women 50+ years^1^: 1.3 (0.9, 1.7)	Age, education, smoking, and regularity of menstruation
				Total number of menstrual cycles (among women 55+ years in a natural menopause): increased risk^1^	
				Length of menstrual cycle, days (<26, 26-29, 30-33, >33): 2.2 (1.3, 3.7), 1.6 (1.0, 2.7), 1.6 (1.0, 2.6), 1.0	
(20)	Wu-Williams AH, Dai XD, Blot W, et al., 1990	Harbin, Shenyang, China	965/959	Age at natural menopause, years (<45, 45-49, 50-54, 55+): 1.0, 1.7 (1.2, 2.4), 1.3 (0.9, 1.8), 1.7 (1.0, 3.2)	Age, education, personal smoking, and study area
(21)	Liao Ml, Wang JH, Wang HM, et al., 1996	Shanghai, China	162^1^/19^3^/187	Total number of menstrual cycles^3^: case mean = 453.2, control mean = 413.3 p < 0.05^4^	Not applicable
				Menstrual flow, days^1^: case mean=4.85, controls mean = 5.29 p < 0.01^4^	
(22)	Zhou B, Wang T, Guan P, Wu JM, 2000	Shenyang, China	72^1^/72	Age at menarche, years (<16, 16+): 0.64 (0.31, 1.30), 1.0	None
				Length of menstrual cycle, days (<30, 30+): 1.12 (0.40, 3.10), 1.0	
				Number of live births (0-1, 2, 3, 4+): 1.0, 0.97 (0.12, 4.91), 1.39 (0.23, 8.52), 2.32 (0.45, 12.02) p for trend = 0.04	Income, eye irritation from smoke, history of lung cancer
	Present study	Gansu Province, China	109/435	Age at menarche, years (<15, 15-16, 17+): 1.0, 0.52 (0.3, 0.88), 0.54 (0.31, 0.96) p for trend = 0.015	Age, prefecture Further adjustment for socioeconomic status, active smoking, environment tobacco smoke among non-smokers, amount of coal, and previous pulmonary diseases had no effect
				Age at natural menopause, years (<45, 45-49, 50+): 1.0, 0.95 (0.49, 1.86), 0.39 (0.17, 0.92) p for trend = 0.074	
				Age at first live birth, years (≤18, 19-22, 23+): 1.0, 1.53 (0.89, 2.64), 1.46 (0.75, 2.84) p for trend = 0.980	

^1^ Adenocarcinoma.

^2^ Odds ratio (95% confidence interval).

^3^ Squamous carcinoma.

^4^ Test for homogeneity of variance.

A biological involvement of sex hormones in pathogenesis of lung cancer, particularly adenocarcinoma, seems plausible based on the presence of specific estrogen and progesterone receptors in normal and some malignant lung tissues,^7^^-^^10^ animal experiments,^11^^-^^12^ and effects of sex hormones on other non-target tumors.^13^^-^^15^ However, the exact mechanism and whether it is similar to other hormone-dependent tumors is unknown. If sex hormones indeed influence the development of lung cancer, then the association between reproductive factors and lung cancer is likely to be weak, which might explain the inconsistency of the epidemiologic results given the limited number of studies. In addition, any association of reproductive factors may occur with a particular histological type or pattern of receptor expression in lung cancer. If so, then risk estimates would be diluted when all lung cancer types are analyzed together as in this study. Finally, hormones may not be carcinogenic themselves but rather modify the effects of other exposures important in the development of lung cancer.

## CONCLUSION

In summary, the results of this study suggest a possible role of female hormones in the etiology of lung cancer. However, given the limitations of our study, these results warrant cautious interpretation.
